# Design rules for light-emitting electrochemical cells delivering bright luminance at 27.5 percent external quantum efficiency

**DOI:** 10.1038/s41467-017-01339-0

**Published:** 2017-10-30

**Authors:** Shi Tang, Andreas Sandström, Petter Lundberg, Thomas Lanz, Christian Larsen, Stephan van Reenen, Martijn Kemerink, Ludvig Edman

**Affiliations:** 1 The Organic Photonics and Electronics Group, Department of Physics, Umeå University, Umeå, SE-901 87 Sweden; 2 LunaLEC AB, Linnaeus väg 24, Umeå, SE-901 87 Sweden; 3 Complex Materials and Devices, Department of Physics, Chemistry and Biology (IFM), Linköping University, Linköping, SE-58183 Sweden

**Keywords:** Electronic devices, Organic LEDs

## Abstract

The light-emitting electrochemical cell promises cost-efficient, large-area emissive applications, as its characteristic in-situ doping enables use of air-stabile electrodes and a solution-processed single-layer active material. However, mutual exclusion of high efficiency and high brightness has proven a seemingly fundamental problem. Here we present a generic approach that overcomes this critical issue, and report on devices equipped with air-stabile electrodes and outcoupling structure that deliver a record-high efficiency of 99.2 cd A^−1^ at a bright luminance of 1910 cd m^−2^. This device significantly outperforms the corresponding optimized organic light-emitting diode despite the latter employing calcium as the cathode. The key to this achievement is the design of the host–guest active material, in which tailored traps suppress exciton diffusion and quenching in the central recombination zone, allowing efficient triplet emission. Simultaneously, the traps do not significantly hamper electron and hole transport, as essentially all traps in the transport regions are filled by doping.

## Abstract (web summary)

Cost-efficient light-emitting electrochemical cells can deliver unique functions, but suffer because strong brightness has only been obtained at modest efficiency. Tang et al. report on rationally designed devices that are both bright and efficient, thus representing a major step towards commercialization.

## Introduction

The light-emitting electrochemical cell (LEC) is a thin-film and area-emitting device that recently has been integrated into, or deposited onto, a broad range of surfaces, including plastic^1–3^, paper^4^, textile^5–7^, and metal^8^. In its common manifestation, an LEC features a single-layer air-stabile active material sandwiched between two air-stabile electrodes; and as such it can be fabricated entirely from environmentally friendly raw materials^3,9,10^ using cost-efficient and scalable solution-based methods^8,11,12^. It can also deliver essentially any emission color, as dictated by the energy gap of the constituent emissive organic semiconductor in the active material. It is the combination of these intrinsic advantages that separates the LEC from commercially introduced emission technologies such as the light-emitting diode (LED), the organic LED (OLED), and the light-emitting capacitor, and which promises the emergence of important and paradigm-shifting emissive applications—such as point-of-care diagnostic and treatment devices, emissive and conformable fabrics^5,6^, and low-cost and low-voltage illumination panels^2,8^. However, a critical challenge with LEC devices is that it has not been possible to achieve high-efficiency operation at significant luminance^13–15^.

A characteristic feature of the LEC, which distinguishes it from the more common-place OLED, is the presence of mobile ions in the active material^16–18^. When a voltage is applied between the electrodes, these ions redistribute to enable in-situ electrochemical doping of the organic semiconductor: *p*-type at the anode and *n*-type at the cathode; after a turn-on time, a *p*-*n* junction has formed in the active material^19,20^. This dynamic doping mode allows for large and balanced electron and hole currents, and a high recombination rate of electrons and holes into excitons. Unfortunately, it also brings a challenge, as the exciton will be quenched when it impinges upon an electron or a hole (commonly termed polarons in organic semiconductors), and this exciton–polaron quenching is severe in LEC devices—to such a degree that it has been argued that high brightness and high efficiency are mutually exclusive^15,21^.

The first LEC comprised a conjugated polymer (CP) as the emissive organic semiconductor^19^, and the current state-of-the-art CP-LEC delivers a current efficacy of 14.6 cd A^−1^ at a luminance of 112 cd m^−2^.^22^ Note that we only consider reports with a luminance surpassing 100 cd m^−2^ as our focus is on realizing practical LECs featuring strong luminance at high efficiency. The second major class of LEC devices comprise an ionic transition metal complex (iTMC) as the emissive organic semiconductor, and because this material group can harvest both singlet and triplet excitons, the efficiency ceiling is higher^23^. A generic problem with iTMC-LECs is instead that the characteristic long lifetime of the triplet exciton translates into a long diffusion distance for mobile triplets, which in combination with a high polaron concentration results in severe exciton-polaron quenching. The best iTMC-LEC performance as-of-today, a current efficacy of 28.2 cd A^−1^ at a brightness of 750 cd m^−2^, was obtained using non steady-state pulsed-current driving^24^, whereas conventional constant-bias driving results in a lower peak performance^25–29^.

The issue of exciton-polaron quenching has been effectively addressed in triplet-emitting OLEDs through the design of a sophisticated device architecture comprising a multitude of different layers, where each layer has a specific task and must feature an exact thickness on the nm-level^30,31^. The central emitting layer is of a host–guest character, where the exciton is trapped on a triplet-emitting guest. This complex and exact device architecture is fabricated by sequential vapor deposition under high vacuum, and is as such not compatible with the simplicity of the LEC concept. Nevertheless, a number of recent attempts at much simpler host–guest LEC architectures have resulted in a wide variety of emission colors, but none of these devices has surpassed an efficiency of 10 cd A^−1^ at a luminance larger than 100 cd m^−2^ during steady-state operation^32–46^. Thus, the critical issue of delivering efficient operation at high luminance from an LEC device remains^13–15^.

Here, we report a general method for the attainment of bright and efficient LEC devices. The identified key criteria comprise the employment of a designed multi-component host:guest:electrolyte active material, in which traps with tailored depth and concentration suppress exciton diffusion and quenching in the central recombination zone, allowing efficient triplet emission. Simultaneously, the traps do not significantly hamper electron and hole transport, as essentially all traps in the transport regions are filled by doping. Such designed LEC devices with air-stabile electrodes are demonstrated to deliver a high luminance well above 1000 cd m^−2^ at a high efficiency of 100 cd A^−1^. We further report on the scalability of the approach through the solution-based spray-coating fabrication of efficient large-area devices and universality via the demonstration of four different efficient and bright host–guest systems.

## Results

### Core concept and simulations

**Fig. 1:**
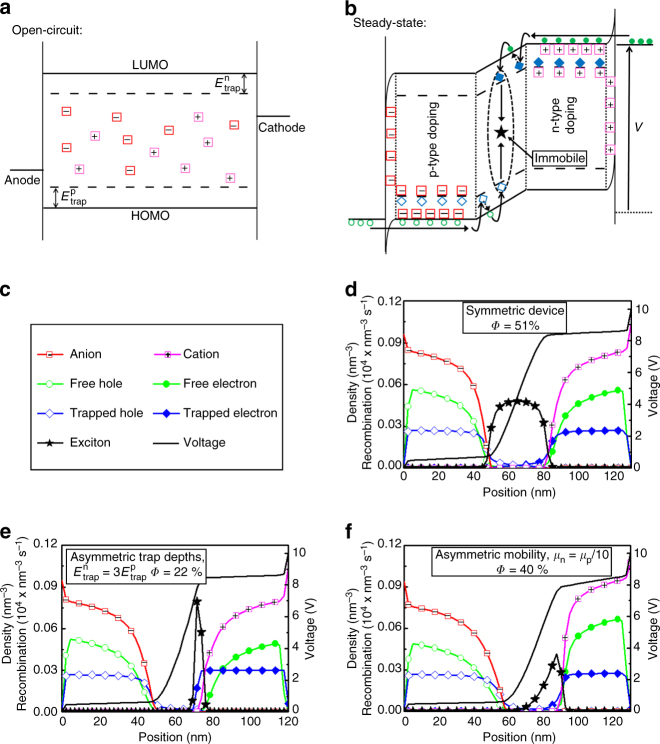
Schematics and steady-state simulation profiles of host–guest light-emitting electrochemical cells. Schematics of the designed host–guest LEC **a** at open circuit and **b** during steady-state operation, with the energy levels of the host and guest indicated by the solid and dashed lines, respectively, and the symbols identified in panel **c**. **d** The simulated steady-state concentration and voltage profiles for a symmetric host–guest LEC. The same device with **e** a deepened electron-trap level or **f** a decreased electron mobility in the host. The insets present the calculated quantum efficiency, assuming 100% photoluminescence quantum efficiency of the guest emitter and perfect outcoupling

Here, we solve the efficiency vs. brightness issue in LECs by blocking the exciton diffusion that precedes quenching in conventional LECs through the introduction of tailored and balanced traps in the active layer. To circumvent the undesired side effect of a reduced conductivity of mobile charges in the *p*- and *n*-doped regions due to the very same traps, the trap and ion concentrations are balanced in such a way that in the *p*- and *n*-type doped regions the traps are filled by the doping, leading to a nearly trap-free transport. Figure 1a shows a schematic of our target host–guest LEC, with the guest energy levels (dashed lines) symmetrically positioned within the energy gap of the host (solid lines). Figure 1b displays its steady-state operation when the electrolyte ions have assisted with the doping (filling) of essentially all of the guest traps, as well as some of the host sites, in distinct doping regions next to the electrodes to facilitate for a large current; importantly, the central *p*-*n* junction is left doping-free to allow for efficient emission from immobile triplet excitons localized on guest traps. In this target scenario, a high and balanced electron/hole current can be maintained while detrimental exciton-polaron interactions are kept to a minimum by suppressing exciton diffusion.

To investigate whether this target LEC operation is attainable, and, if so, to establish rational device design guidelines, we performed numerical simulations on a variety of different host–guest LEC architectures. The details of the modeling procedure and the parameter selections are described in the Methods section. Figure 1d presents the steady-state concentration and voltage profiles for the best performing of the simulated devices, with the symbols identified in Fig. 1c. All ions have performed electrochemical doping, as evidenced by that the ion concentration (squares) is equal to the sum of the free polarons on the host (circles) and the trapped polarons on the guest (diamonds) throughout the active material. The only exception is next to the electrode interfaces, where electric-double layers have formed to compensate for the significant energy difference (1 eV) between the Fermi level of the injecting electrode and the accepting energy level of the primary transport material, i.e., the host—this characteristic feature of LECs that allows the use of air-stable electrodes is not affected by the introduction of traps.

The coupled ion/polaron concentration profile defines a distinct doping structure for the 130-nm thick device, with 45-nm-thick *p*-type doping and *n*-type doping regions bridging the *p*-*n* junction, where the total polaron concentration remains very low. We find that essentially all guest traps (*c*_trap_ = 0.03 nm^−3^) are filled in the doping regions, while the emission is observed to originate solely from immobile excitons (stars) positioned on dispersed guest molecules in the *p*-*n* junction. The device thus accomplished the targeted doping and emission profiles, as schematically indicated in Fig. 1b, and the in silico functionality of the concept is evidenced by the high simulated electron-to-photon quantum efficiency of *Φ* = 51% at a large current density of *j = *4.5 mA cm^−2^.

The second important outcome of the simulation study is that the performance of the host–guest LEC is highly sensitive to the selection and symmetry of a number of controllable device parameters. Figure 1e and f presents the concentration and voltage profiles for devices with asymmetric hole and electron trap depths and asymmetric hole and electron mobilities in the host, respectively, as specified in the insets. When the electron-trap depth is increased (Fig. 1e), or the electron mobility is lowered (Fig. 1f), the exciton profile is strongly shifted towards the *n*-type doping region, resulting in a notably increased overlap between the electron–polaron and exciton populations. The concomitant increase in exciton–polaron quenching is manifested in that the efficiency drops to *Φ* = 22% (at *j* = 2.8 mA cm^−2^) for the asymmetric trap-depth device and to *Φ* = 40% (at *j* = 1.8 mA cm^−2^) for the asymmetric mobility device.

### Material characterization and selection

**Fig. 2:**
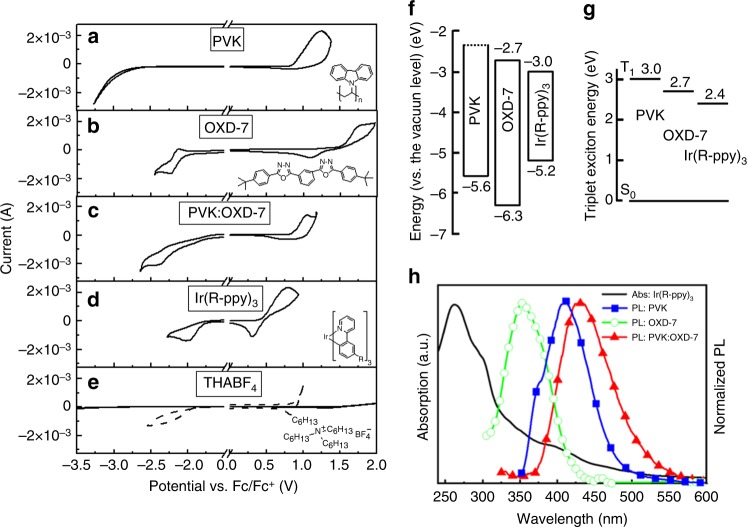
Cyclic voltammetry (CV) and optical measurements of device materials. **a**–**d** CV traces recorded on thin films of the different host and guest constituents, with the corresponding chemical structure displayed in the right inset. **e** CV for the THABF_4_ electrolyte (solid line) and a TMPE-OH:LiCF_3_SO_3_ electrolyte (dashed line). **f** The electron-energy levels of the host and guest materials, as extracted from the CV data. The (dotted) LUMO level of PVK could not be measured with CV, and was instead estimated from the absorption data. **g** The triplet energies of the host and guest compounds. **h** The absorption spectrum of the guest compound (solid black line) and the PL spectra of the three host materials

With the inspiring simulation result at hand, we turn to the identification of appropriate LEC materials. First, it is fundamental that the target LEC comprise host and guest compounds that can be electrochemically doped, while the electrolyte should be electrochemically inert within the voltage range spanned by the electrochemical doping reactions. Figure 2a–e presents a systematic cyclic voltammetry (CV) interrogation of a number of commercially available candidate compounds, with their corresponding chemical structure displayed in the inset. We find that the poly(9-vinylcarbazole) (PVK) host only features *p*-type doping capacity, as implied by the lack of a reversible reduction event; this conclusion is in agreement with direct optical probing of planar PVK-based LECs^37^. The 1,3-bis[2-(4-tert-butylphenyl)−1,3,4-oxadiazo-5-yl]benzene (OXD-7) host and the PVK:OXD-7 blend host can, in contrast, be both electrochemically *p*-type and *n*-type doped, and are thus qualified as appropriate LEC host materials. A comparison of the top three CV traces reveals that for the blend host it is PVK that is (preferentially) *p*-type doped and OXD-7 that is *n*-type doped.

Figure 2d shows that the tris[2-(5-substituent-phenyl)-pyridinato]iridium(III) (Ir(R-ppy)_3_) guest compound displays significant *p*- and *n*-type doping capacity, and that both its doping reactions are thermodynamically preferable in comparison to all three hosts. Supplementary Figure 1 also reveals that it is primarily the Ir(R-ppy)_3_ guest that is *p*- and *n*-type doped in a PVK:OXD-7:Ir(R-ppy)_3_ blend film. We further find that the THABF_4_ ionic liquid (Fig. 2e, solid line) is fit for the task of electrolyte, as it is electrochemically silent over the entire voltage range spanned by the *p*- and *n*-type doping potentials of the host and guest compounds. This is in contrast to, e.g., TMPE-OH:LiCF_3_SO_3_, the electrolyte of choice in recent state-of-the art CP-LECs^47^, which electrochemical stability is insufficient (Fig. 2e, dashed line). This finding is confirmed in Supplementary Fig. 2, which demonstrates that it is possible to n-type dope OXD-7 and *p*-type dope PVK with the THABF_4_ electrolyte, but not with the TMPE-OH:LiCF_3_SO_3_ electrolyte.

Figure 2f presents the electron-energy diagram for the host and guest compounds, as extracted from the CV data (with the exception of the LUMO of PVK that was derived from a combined absorption/CV measurement). It clearly illustrates that the exciton, as well as both the hole and electron, can be trapped on the Ir(R-ppy)_3_ guest when positioned in any of the three host matrices, i.e., PVK, OXD-7, and the PVK:OXD-7 blend. The triplet diagram in Fig. 2g shows that host-to-guest Dexter transfer is feasible for all host systems^48,49^, while the significant spectral overlap of the host photoluminescence (PL) and the guest absorption displayed in Fig. 2h implies that host-to-guest Förster resonance transfer can be efficient for all three host–guest systems. The conclusion is thus that the exciton can be efficiently funneled to the dispersed guest molecules using a combination of Förster transfer, Dexter transfer and charge trapping. After arriving at a guest molecule the exciton will not move any further, provided the guest concentration is sufficiently low to suppress exciton diffusion between different guest molecules.

### Optoelectronic characterization and performance discussion

**Fig. 3:**
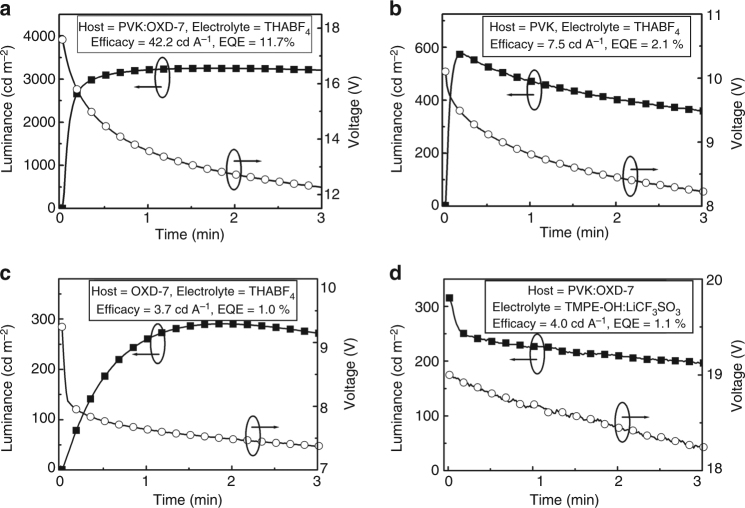
Device performance of host–guest light-emitting electrochemical cells. The temporal optoelectronic response of ITO/PEDOT:PSS/Host:Ir(R-ppy)_3_: Electrolyte/Al LECs with the host and electrolyte selection defined in the panels **a**–**d**. All devices featured identical host:guest:electrolyte mass stoichiometry, active-material thickness (130 nm), and drive current density (*j* = 7.7 mA cm^−2^)

The characterized materials were used for the fabrication of LEC devices, comprising an indium-tin-oxide (ITO)/poly(3,4-ethylenedioxythiophene):poly(styrene sulfonate) (PEDOT:PSS) anode and an Al cathode. The transient optoelectronic data in Fig. 3a–d are recorded on pristine devices with the same active-material mass stoichiometry (host:guest:electrolyte = 64.6:29.0:6.4) and thickness (*d* = 130 nm) and which were driven by the same current density, *j* = 7.7 mA cm^−2^. The distinguishing characteristic was the selection of the host and electrolyte, as identified in the figure insets. Supplementary Figure 3a shows that all four devices emitted green light (*λ*_peak_ = 520 nm) from the guest compound, indicating complete energy transfer from host to guest (Fig. 2f–h).

Importantly, the PVK:OXD-7 blend-host device in Fig. 3a exhibited a record-high LEC performance, delivering a strong luminance of 3250 cd m^−2^ at a current efficacy of 42.2 cd A^−1^; the latter corresponds to an external quantum efficiency (EQE) = 11.7%. Note that all presented efficiency values throughout the manuscript are reported at maximum luminance. The pristine blend-host device turned on fast and reached 100 cd m^−2^ in <2 s and 3000 cd m^−2^ within 25 s, and maintained a high luminance of above 1000 cd m^−2^ for >3 h of continuous operation.

However, we find that the host–guest LEC performance is highly sensitive to the material selection, as summarized in Supplementary Table 1. The PVK-host device in Fig. 3b delivered a peak brightness of 575 cd m^−2^ at an efficiency of 7.5 cd A^−1^, and the luminance began to drop already after 10 s of operation. We attribute its comparatively poor performance to that the PVK host can only support *p*-type doping (Fig. 2a), and that electrochemical side reactions accordingly will take place at the cathodic interface during device operation. The even lower performance of the blend-host device with TMPE-OH:LiCF_3_SO_3_ as the electrolyte instead of THABF_4_ (Fig. 3d) is attributed to the insufficient electrochemical stability window of the electrolyte, which will effectively prohibit both *p*- and *n*-type doping of the host compounds (Fig. 2e and Supplementary Figure 2). The absence of conductivity-enhancing electrochemical doping reactions of the host compounds is further manifested in that the luminance decreases monotonically with increasing operational time.

The explanation for the one order of magnitude lower efficiency for the OXD-7 device in Fig. 3c in comparison to the blend-host device in Fig. 3a is at first glance less obvious. The OXD-7 host compound can be both *p*- and *n*-type doped (Fig. 2b) and the employed THABF_4_ electrolyte is electrochemically stable (Fig. 2e). However, with the results of the simulation study at hand (Fig. 1c–f), we instead turn our attention to other material properties, in particular trap depths and charge carrier mobilities.

The nominal electron-trap depth $( {E_{{\mathrm{trap}}}^{\mathrm{n}}} )$ is equal to the difference between the (lowest) LUMO level of the host(s) and the LUMO of the guest, and the nominal hole-trap depth $( {E_{{\mathrm{trap}}}^{\mathrm{n}}} )$ is given by the difference between the (highest) HOMO of the host(s) and the HOMO of the guest (Fig. 1a). Using the electron–energy diagram in Fig. 2f, we find that for the blend-host LEC $E_{{\mathrm{trap}}}^{\mathrm{n}}$ = 0.32 eV and $E_{{\mathrm{trap}}}^{\mathrm{p}}$ = 0.38 eV; for the single-host OXD-7 LEC, we obtain $E_{{\mathrm{trap}}}^{\mathrm{n}}$ = 0.32 eV and $E_{{\mathrm{trap}}}^{\mathrm{p}}$ = 1.06 eV. In other words, while the trap depths for electron and hole transport in the blend-host LEC are rather symmetric, they are notably asymmetric in the OXD-7 LEC. Moreover, the PVK:OXD-7 blend-host features a balanced electron and hole mobility, while the single-host OXD-7 material is an *n*-type material with a distinctly higher electron mobility^50^. The simulation results demonstrated that an asymmetry in the trap depth (Fig. 1e), as well as an asymmetry in the electron and hole mobility (Fig. 1f), will result in an increase in detrimental exciton–polaron interactions, with a concomitant significant drop in the efficiency. Thus, the conclusion from both experiments and simulations is that it is paramount to pay attention to the symmetry of the trap-depth and mobility parameters when designing a high-performance host–guest LEC.

Within the project, we have investigated a wide range of different host–guest combinations, and been able to identify three additional host–guest LECs that feature high efficiency at strong luminance; see Supplementary Fig. 4. With a green-emitting tris[2-(p-tolyl)pyridine]iridium (III) (Ir(mppy)_3_) guest, and the same PVK:OXD-7 blend host and THABF_4_ electrolyte, we recorded a current efficacy of 24.6 cd A^−1^ (EQE = 6.9%) at 1890 cd m^−2^, and with a red-emitting tris[2-(4-n-hexylphenyl)quinoline]iridium (III) (hex-Ir(piq)_3_) guest an efficiency of 3.9 cd A^−1^ (EQE = 4.9%) at 302 cd m^−2^ was measured. With a different TH123:TH105 host blend, and the Ir(R-ppy)_3_ guest, we detected green emission with an efficiency of 27.4 cd A^−1^ (EQE = 7.6%) at 2100 cd m^−2^. It is interesting that these three high-performing host–guest LECs also feature bipolar doping, balanced mobility, and reasonably balanced trap depths. A comparison does however reveal that the PVK:OXD-7:Ir(R-ppy)_3_ LEC displayed the best performance (and the most symmetric trap depths), and in the following we have therefore opted to stay with this system. A summary of relevant device and material data is available in Supplementary Table 1.

### Optimization and scale up

**Fig. 4:**
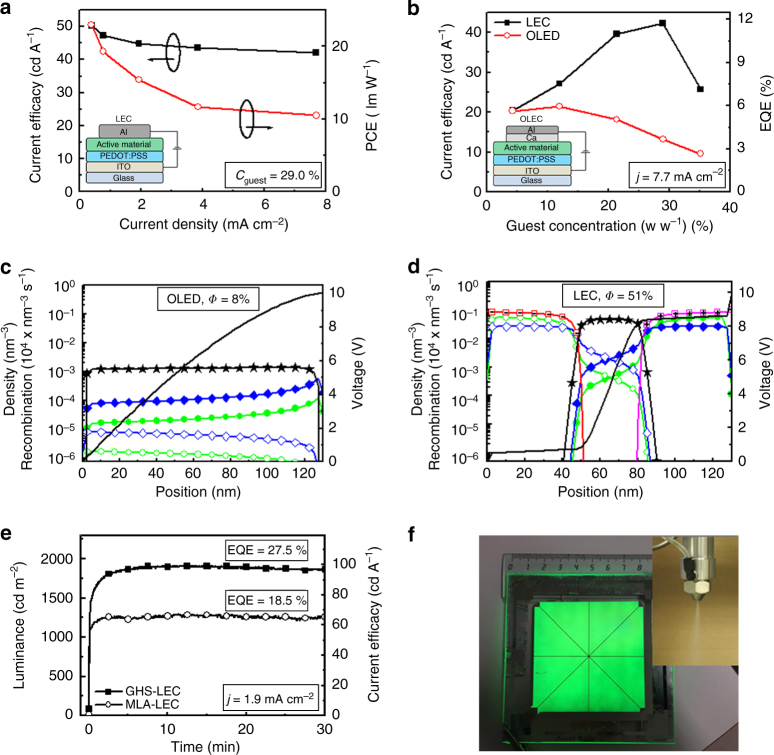
Device optimization and large-area fabrication. **a** The measured current efficacy (solid black squares) and power conversion efficacy (open red circles) as a function of the current density for the host–guest LEC, with a guest concentration of 29% and with the PVK:OXD-7 blend host. **b** The current efficacy (left) and external quantum efficiency (right) as a function of guest concentration for the ITO/PEDOT:PSS/PVK:OXD-7:Ir(R-ppy)_3_:THABF_4_/Al LEC (solid black squares) and a ITO/PEDOT:PSS/PVK:OXD-7:Ir(R-ppy)_3_/Ca/Al OLED (open red circles). The devices featured the same active-material thickness (130 nm) and were driven by *j* = 7.7 mA cm^−2^. **c** The simulated steady-state concentration profiles for a host–guest OLED, with realistic injection barriers for electrons (0.2 eV) and holes (0.5 eV). **d** The simulated steady-state concentration profiles for a host–guest LEC with an injection barrier of 1 eV for both electron and hole injection. Both devices feature symmetric trap depths and mobilities, and the symbols are identified in Fig. 1c. **e** The initial evolution of the luminance (left axis) and the current efficacy (right axis) of pristine ITO/PEDOT:PSS/PVK:OXD-7:Ir(R-ppy)_3_:THABF_4_/Al LECs featuring a thin film comprising a hexagonal array of hemispherical microlenses (MLA-LEC, open circles) or a glass half sphere (GHS-LEC, solid squares) as the outcoupling structure. Both LECs were driven by a current density of *j* = 1.9 mA cm^−2^. **f** The uniform green light-emission from a 45 cm^2^ large-area host–guest LEC fabricated by spray-sintering under ambient air. Inset: photograph depicting the airbrush performing spray-sintering deposition

A further demonstration of the suppressed exciton-polaron quenching in a well-designed host–guest LEC is provided by the efficiency–current plot in Fig. 4a. If such detrimental interactions are prominent, these will be manifested in a significant drop of the current efficacy with increasing current. Here we find that the current efficacy only dropped from 50.4 cd A^−1^ (EQE = 14.0%) to 42.2 cd A^−1^ (EQE = 11.7%), when the current density increased by a factor of 20 from 0.38 to 7.7 mA cm^−2^, which implies that polaron–exciton quenching indeed is relatively minor. Note that the highest current efficacy of 50.4 cd A^−1^ was recorded at a reasonable luminance of 193 cd m^−2^. We further observe that the power conversion efficacy features a stronger dependence on the drive current than the current efficacy, which is a reflection of a lowering of the drive voltage with decreasing current. The symmetric host–guest LEC required a drive voltage of ~9 V in order to reach 1000 cd m^−2^ at steady state, and the overpotential (i.e., the voltage exceeding the effective energy-gap potential of the host compounds) is dropping over the undoped, and therefore poorly conducting, *p*-*n* junction region, as visualized in the voltage profile in Fig. 1d. It is anticipated that the conductivity of the *p*-*n* junction region in the symmetric host–guest LEC will be lower than in a conventional LEC, because of the existence of non-filled traps in the junction region. Nevertheless, the drive voltage is observed to drop with decreasing current and luminance to reach below 5 V at 100 cd m^−2^; see Supplementary Fig. 5.

Figure 4b presents the efficiency as a function of guest concentration for the host–guest LEC and a nominally similar OLED, void of the electrolyte and with low-work function Ca instead of Al as the cathode. The schematic LEC and OLED device structures are displayed in the insets of Fig. 4a and b, respectively. Both devices display green emission from the guest compound for all concentrations (Supplementary Fig. 3b), in agreement with the demonstrated efficient host–guest energy transfer. Interestingly, the LEC outperforms the OLED over the entire guest-concentration range, with the difference being largest at the higher guest concentrations. The peak efficiency of the LEC is 42.2 cd A^−1^ (11.7%), whereas the OLED peaked at 21.7 cd A^−1^ (6.0%) at a three times lower guest concentration.

To investigate the cause for the differences in performance, we present modeling results for a host–guest OLED with device-realistic injection barriers for electrons and holes of 0.2 eV and 0.5 eV, respectively, (Fig. 4c) and for a host–guest LEC with a larger injection barrier of 1.0 eV for both electron and hole injection (Fig. 4d). Both devices featured symmetrical trap depths and mobilities, but in order to make the current density similar the absolute mobility was set higher for the OLED, which is reasonable in view of the absence of the electrolyte in the OLED. The absence of mobile ions in the OLED is manifested in much lower electron and hole concentrations (as there is no ion-induced screening of the electronic space charge) and a lack of electric double-layer formation at the electrode interfaces. Two more indirect consequences are that the excitons are evenly distributed, and the voltage gradient relatively constant, throughout the entire OLED active material, whereas they are well confined to the central *p*-*n* junction in the LEC device. Importantly, while the simulated OLED delivers a modest electron-to-photon efficiency of *Φ* = 8%, the LEC features a much higher *Φ* = 51% at approximately the same current density (*j*_OLED_ = 3.4 mA cm^−2^, *j*_LEC_ = 4.5 mA cm^−2^).

A significant fraction of the photons are trapped within the planar device structure by total internal reflection, and in order to extract also these photons we have attached two different outcoupling structures onto the transparent substrate: a flexible thin film comprising a hexagonal array of hemispherical microlenses as the surface structure (MLA-LEC), and an index-matched glass half-sphere (GHS-LEC). The transient optoelectronic response is presented in Fig. 4e: with the MLA-LEC we measured a current efficacy of 66.8 cd A^−1^ at 1285 cd m^−2^ and with the GHS-LEC, we reached 99.2 cd A^−1^ at 1910 cd m^−2^. The latter corresponds to an impressive external quantum efficiency of 27.5%. For the same device we measured a stress lifetime of 320 h above 100 cd m^−2^ in Supplementary Fig. S6a. The corresponding data at a higher current density of *j* = 7.7 mA cm^−2^ are presented in Supplementary Fig. 6b, and we mention that the GHS-LEC delivered a strong luminance of 7200 cd m^−2^ at a retained high efficiency of 92.8 cd A^−2^; a summary of the data is available in Supplementary Table 2.

We have also worked on improving the stability of the symmetric host–guest LEC system. Our hypothesis, as inspired by earlier experimental results^51^, is that a high local exciton–polaron interaction rate will result in detrimental side reactions, and that it is this rate that is effectively dictating the operational lifetime of a well-designed LEC. We have again turned to modeling to direct our experimental activity, and Supplementary Fig. 7 demonstrates that it is possible to control the polaron distributions and the effective thickness of the *p*-*n* junction by the ion concentration; and as a consequence the exciton–polaron interaction rate. By varying the ion concentration, we could identify an ion concentration at which the local exciton–polaron interaction rate is minimized, while a facile and balanced injection and transport of electrons and holes is maintained. We find that this sweet-spot ion concentration is three times lower than the ion concentration employed in the device presented in Fig. 1d. To our satisfaction, this simulation result could be translated to experiments, as a corresponding lowering of the ion concentration in symmetric host–guest LEC devices resulted in an improvement of the operational lifetime by a factor of four, while the drop in the luminance and efficiency was found to be relatively marginal. By further implementing a pulsed-current driving, as introduced by Bolink and co-workers^52, 53^, we found that the operational lifetime could be improved by an additional 60%, presumably since the polaron and exciton populations are further separated when the device is never allowed to reach steady state. These performance data are summarized in Supplementary Table 3.

All devices up to this stage have featured a small light-emission area of <0.2 cm^2^ as fabricated by spin-coating, but we have also fabricated large emission-area LECs by more scalable spray-coating (or more specifically spray-sintering)^8^ using an in-house developed automated spray-coating apparatus. The photograph in Fig. 4f shows the uniform green light-emission from such a 45 cm^2^ large-area host–guest LEC, with its 350 nm thick active material deposited by spray-sintering under ambient air, as displayed in the inset photograph. The large-area LEC device was driven with a constant current of *j* = 1.1 mA cm^−2^, and delivered a luminance of 150 cd m^−2^ at a current efficacy of 13 cd A^−1^, despite being void of an outcoupling structure. This result indicates that the introduced approach to high-efficiency, high-luminance LEC operation is relevant also for practical low-cost and/or large-area device configurations, and as such could pave the way for a wide range of paradigm-shifting and important applications in, for instance, home health care and portable signage where high-brightness operation at low energy consumption and low cost can be a fundamental criterion.

## Discussion

We report on the conceptualization and realization of designed host–guest LECs with air-stabile electrodes and a single-layer active material, which deliver bright green luminance of 1910 cd m^−2^ at a high current efficacy of 99.2 cd A^−1^. The active material comprised rationally selected host, guest, and electrolyte compounds, so that the polarons are primarily confined to distinct and highly conductive doping regions, whereas the triplet excitons are formed and trapped on dispersed guest molecules in a well-defined central *p*-*n* junction region. With this balanced host–guest approach we are able to strongly suppress detrimental exciton–polaron quenching, even at high current density. It is notable that the high-performance LEC is twice as efficient as a nominally identical OLED void of the electrolyte and comprising Ca instead of Al as the cathode. We further demonstrate scalability through the fabrication of efficient large-area host–guest LECs using a home-built spray-sintering apparatus, as well as universality through the attainment of high-efficiency and high-luminance from three other LEC systems fulfilling the same baseline criteria. We finally wish to reinforce that the LEC technology offers unique advantages from a low-cost and low-energy fabrication viewpoint, and we hope that the demonstration of bright LEC devices, with an efficiency closing in on that of state-of-the-art OLEDs, will pave the way for the emergence of sustainable and low-cost emissive devices in important applications such as point-of-care medical diagnosis and treatment.

## Methods

### Materials

The chemical structure of the host materials poly(9-vinycarbazole) (PVK, Sigma-Aldrich) and 1,3-bis[2-(4-tert-butylphenyl)-1,3,4-oxadiazo-5-yl]benzene (OXD-7, Lumtec) are presented in the inset of Fig. 2a, b. Other investigated host compounds include the commercially available materials Triplet Host 123 (TH123, Merck) and Triplet Host 105 (TH105, Merck). A wide range of commercially available guest compounds were investigated, including tris[2-(5-substituent-phenyl)-pyridinato]iridium(III) (Ir(R-ppy)_3_, Merck, see Fig. 2d), tris[2-(p-tolyl)pyridine]iridium(III) (Ir(mppy)_3_, Lumtec), and tris[2-(4-n-hexylphenyl)quinoline]iridium(III) (Hex-Ir(piq)_3_, Lumtec); see insets in Supplementary Fig. 4a and b. The investigated electrolytes are tetrahexylammonium tetrafluoroborate (THABF_4_, Sigma-Aldrich, see Fig. 2e) and LiCF_3_SO_3_ (Sigma-Aldrich) dissolved in hydroxyl-capped trimethylolpropane ethoxylate (TMPE-OH, *M*_*w*_ = 450 g mol^−1^, Sigma-Aldrich). All of the materials were used as received. The master solutions were prepared by dissolving the constituent material in chlorobenzene at a concentration of 15 mg ml^−1^ (PVK), 30 mg ml^−1^ (OXD-7), 20 mg ml^−1^ (PVK:OXD-7), 20 mg ml^−1^ (TH123:TH105), 10 mg ml^−1^ (THABF_4_), and 10 mg ml^−1^ (TMPE-OH:LiCF_3_SO_3_). The master solutions were stirred on a magnetic hot plate at 343 K for at least 5 h before further processing.

### Material characterization

Cyclic voltammetry (CV) was carried out with an Autolab PGSTAT302 potentiostat driven by the GPES software. The working electrode comprised the material-under-study coated on a Au-covered glass substrate, a Pt rod was the counter electrode, a Ag wire was the quasi-reference electrode, and 0.1 M tetrahexylammonium tetrafluoroborate (THABF_4_, Sigma-Aldrich) in anhydrous CH_3_CN was the electrolyte. Directly after each CV scan, a calibration scan was run with a small amount of ferrocene added to the electrolyte. All CV potentials are reported vs. the ferrocene/ferrocenium ion (Fc/Fc^+^) reference potential. The reduction/oxidation onset potentials are defined as the intersection of the baseline with the tangent of the current at the half-peak-height. The energy structure (i.e., the HOMO and LUMO levels) of the material-under-study was derived using the equation *E*_VL_ = −*e*·(4.8 V + *V*_Fc/Fc+_). The CV sample preparation and characterization were executed in a N_2_-filled glove box ([O_2_] < 1 p.p.m., [H_2_O] < 0.5 p.p.m.). The absorption (UV-3100 spectrophotometer, Shimadzu) and photoluminescence (PL; FP-6500 spectrofluorometer, JASCO) measurements were carried out on spin-coated thin films on carefully cleaned quartz substrates.

### Device fabrication and characterization

The active-material inks were prepared by blending the host and electrolyte master solutions in a desired mass ratio, and thereafter adding an appropriate amount of the guest compound. The small-area LEC and OLED devices were fabricated by sequentially spin-coating a poly(3,4-ethylenedioxythiophene):poly(styrene sulfonate) (PEDOT:PSS, Clevios P VP AI 4083, Heraeus) ink at 4000 r.p.m. for 60 s and the active-material ink at 2000 r.p.m. for 60 s onto carefully cleaned indium-tin-oxide (ITO) coated glass substrates (20 Ω per square, Thin Film Devices, US). The dry thickness of the PEDOT-PSS and the active material was 40 nm and ≈130 nm, respectively. For the LEC (OLED), a set of four Al (Ca) cathodes was deposited on top of the active material by thermal evaporation at a base pressure below 5 × 10^–4^ Pa through a shadow mask. The light-emission area, as defined by the size of one cathode, was 8.5 × 1.5 mm^2^. All of the above procedures, except for the deposition of the PEDOT:PSS layer, were carried out in two interconnected N_2_-filled glove boxes ([O_2_] < 1 p.p.m., [H_2_O] < 0.5 p.p.m.). The LEC and OLED devices were characterized using a computer-controlled source-measure unit (Agilent U2722A) and a calibrated photodiode, equipped with an eye-response filter (S9219-01, Hamamatsu Photonics), connected to an embedded evaluation board (myRIO-1900, National Instruments) via a current-to-voltage amplifier. The EL spectra were recorded using a calibrated fiber-optic spectrometer (USB2000+, Ocean Optics).

The planar light out-coupling structure comprised hemispherical lenses in a hexagonal pattern on the surface of a 250 µm thick poly(methyl methacrylate) (PMMA) film (Microsharp). The radius and the height of each microlens were 35 and 24.5 µm, respectively. A UV-curable and single-component acrylic adhesive was used for laminating the outcoupling film onto the LEC device. The hemispherical out-coupling structure comprised a half-sphere lens (*d* = 18 mm, Thorlabs), which was mounted onto the LEC with a specialty index-matching oil (Olympus). Both light out-coupling structures featured a refractive index of *n* = 1.5, which matched the glass substrate onto which they were mounted. The light-emission area of the out-coupled LECs was 1.5 × 1.5 mm^2^, and was defined by etching of the ITO anode.

The large-area LECs were fabricated by spray-sintering under ambient air. The original active-material ink was diluted with 80% tetrahydrofurane, and spray-sintered onto a pre-patterned ITO-coated glass substrate (Thin Film Devices, US) maintained at 70 °C by a hotplate. The spray-sintering deposition was executed using an in-house developed, computer-controlled spray box (LunaLEC AB, Sweden), equipped with an internal-mix spray nozzle. The N_2_ gas pressure was set to 450 × 10^3^ Pa, and the ink-feeding rate was 1 ml min^−1^. The spray nozzle was programmed to move back-and-forth over a 10 × 10 cm^2^ area in a raster-like motion, at a height of 6 cm above the substrate, and to stop after 8 completed sweeps (*t* = 190 s). The resulting dry active-material film thickness was 350 nm. The Al top electrode was deposited by thermal evaporation through a shadow mask, defining the 67 × 67 mm^2^ emission area. The luminance was measured with a luminance meter (LS-110, Konica), and the presented luminance is the average from more than six measurements on different spatial locations over the device area. The photograph was recorded by a digital camera (Canon EOS 300D) using an exposure time of 1/120 s and an aperture of f/2.2.

### Modeling

For the numerical simulations, a 2D drift-diffusion model was used as described in ref. ^15^. In brief, the model solves the coupled continuity equations for electronic and ionic charges and Poison’s equation on a 2D grid by forward integration in time until steady-state has been reached. Motion of all charged species is described by the drift-diffusion equation assuming that the Einstein relation holds. Trap levels are implemented as discrete energy levels whose steady-state occupation is determined by Fermi–Dirac statistics. In the absence of traps, electron–hole recombination is described as a Langevin process; with traps present, recombination is described as a Shockley–Read–Hall process. In both cases, the recombination rate constant is $R = q\mu _{\mathrm{R}}/\varepsilon _0\varepsilon _{\mathrm{r}}$, where the recombination mobility *μ*_*R*_ equals the (sum of the) mobility (mobilities) of the mobile carrier(s). In the absence of traps, excitons can diffuse and exciton–polaron quenching is in that case described by a rate constant as:

1 $$k_1\left( t \right) = 4\pi NR_{{\mathrm{eff}}}D\left[ {1 + \frac{{R_{{\mathrm{eff}}}}}{{\left( {\pi D_{ex}t} \right)^{1/2}}}} \right]$$

where *N* is Avogadro’s number divided by 1000, *D*_*ex*_ the exciton diffusion constant and $R_{{\mathrm{eff}}} = 0.676\left( {\frac{{R_0^6}}{{\tau _{\mathrm{D}}^0D}}} \right)^{1/4}$ with *R*_0_ the Förster critical radius and $\tau _{\mathrm{D}}^0$ the excited state lifetime of the donor in the absence of transfer^54^. The competing (desired) process of radiative emission occurs with a rate $k_{{\mathrm{rad}}} = 1/\tau _{\mathrm{D}}^0$. In the presence of traps, exciton diffusion is zero and the ratio of the radiative quantum yields in the presence and absence of quenching is given by

2 $$\frac{{{{\Phi }}_D}}{{{\Phi}_{D}^0}} = 1 - \sqrt {\pi} \gamma \, {\mathrm{exp}}\left( {\gamma ^2} \right)\left[ {1 - {\mathrm{erf}}\left( \gamma \right)} \right]$$

where $\gamma = \frac{{\sqrt {\pi}}}{2}C_{A}\frac{4}{3}\pi R_{0}^3$ and *C*_A_ is the concentration of acceptors expressed in number of molecules per Å^3^. As in the trap-free case, the radiative emission in absence of quenching $\Phi _{D}^{0}$ is calculated as $\Phi _{\mathrm{D}}^{0} = n_{{\mathrm{ex}}}/\tau _{\mathrm{D}}^{0}$ with *n*_ex_ the exciton concentration. The ratio of the radiative quantum yields was used to determine the rate of polaron quenching:

3 $$k_{\mathrm{q}} = k_{{\mathrm{rad}}}\left( {\frac{{\sqrt{\pi}\gamma \, {\mathrm{exp}}\left( {\gamma ^2} \right)\left[ {1 - {\mathrm{erf}}\left( \gamma \right)} \right]}}{{1 - \sqrt {\pi} \gamma \, {\mathrm{exp}}\left( {\gamma ^2} \right)\left[ {1 - {\mathrm{erf}}\left( \gamma \right)} \right]}}} \right)$$

where *k*_rad_ = $$1{/}\tau _{\mathrm{D}}^{0}$$.

For the injection process, a simplified model was used in which hole (electron) injection is only dependent on the energy difference between the electrode Fermi level and the active layer HOMO (LUMO) at the one but closest point to the electrode edge. This injection model leads to an electric double layer thickness that equals the mesh spacing (here 2.5 nm) but otherwise gives an accurate description of the full device behavior, while being numerically efficient and stable. Further details and a (favorable) comparison to more detailed injection models can be found in the Supporting Information of ref. ^55^. The different simulation parameters are presented and discussed in Supplementary Note 1.

### Data Availability

All relevant data are available from the authors upon request.

## Electronic supplementary material

Supplementary Information

## References

[1] Filiatrault HL, Porteous GC, Carmichael RS, Davidson GJE, Carmichael TB (2012). Stretchable light-emitting electrochemical cells using an elastomeric emissive material. Adv. Mater..

[2] Liang JJ, Li L, Niu XF, Yu ZB, Pei QB (2013). Elastomeric polymer light-emitting devices and displays. Nat. Photon..

[3] Yu Z (2011). Highly flexible polymer light-emitting devices using carbon nanotubes as both anodes and cathodes. J. Photon. Energy.

[4] Asadpoordarvish A (2015). Light-emitting paper. Adv. Funct. Mater..

[5] Lanz T (2016). A light–emission textile device: conformal spray-sintering of a woven fabric electrode. Flex. Print. Electron..

[6] Zhang Z (2015). A colour-tunable, weavable fibre-shaped polymer light-emitting electrochemical cell. Nat. Photon..

[7] Moran-Mirabal JM (2007). Electrospun light-emitting nanofibers. Nano Lett..

[8] Sandström A, Asadpoordarvish A, Enevold J, Edman L (2014). Spraying light: ambient-air fabrication of large-area emissive devices on complex-shaped surfaces. Adv. Mater..

[9] Jürgensen N, Zimmermann J, Morfa AJ, Hernandez-Sosa G (2016). Biodegradable polycaprolactone as ion solvating polymer for solution-processed light-emitting electrochemical cells. Sci. Rep..

[10] Matyba P (2010). Graphene and mobile ions: the key to all-plastic, solution-processed light-emitting devices. Acs Nano.

[11] Sandström A, Dam HF, Krebs FC, Edman L (2012). Ambient fabrication of flexible and large-area organic light-emitting devices using slot-die coating. Nat. Commun..

[12] Hernandez-Sosa G (2014). The compromises of printing organic electronics: a case study of gravure-printed light-emitting electrochemical cells. Adv. Mater..

[13] Meier SB (2014). Light-emitting electrochemical cells: recent progress and future prospects. Mater. Today.

[14] Tang S, Edman L (2016). Light-emitting electrochemical cells: a review on recent progress. Top. Curr. Chem..

[15] van Reenen S, Janssen RAJ, Kemerink M (2015). Fundamental tradeoff between emission intensity and efficiency in light-emitting electrochemical cells. Adv. Func. Mater..

[16] Leger JM (2008). Organic electronics: the ions have it. Adv. Mater..

[17] Slinker JD (2007). Direct measurement of the electric-field distribution in a light-emitting electrochemical cell. Nat. Mater..

[18] deMello JC, Tessler N, Graham SC, Friend RH (1998). Ionic space-charge effects in polymer light-emitting diodes. Phys. Rev. B.

[19] Pei QB, Yu G, Zhang C, Yang Y, Heeger AJ (1995). Polymer light-emitting electrochemical-cells. Science.

[20] AlTal F, Gao J (2016). High resolution scanning optical imaging of a frozen polymer p-n junction. J. Appl. Phys..

[21] Li XY, Gao J, Liu GJ (2013). Reversible luminance decay in polymer light-emitting electrochemical cells. Appl. Phys. Lett..

[22] Mindemark J (2016). High-performance light-emitting electrochemical cells by electrolyte design. Chem. Mater..

[23] Lee JK, Yoo DS, Handy ES, Rubner MF (1996). Thin film light emitting devices from an electroluminescent ruthenium complex. Appl. Phys. Lett..

[24] Tordera D (2013). Low current density driving leads to efficient, bright and stable green electroluminescence. Adv. Energy Mater..

[25] Bolink HJ (2006). Stable single-layer light-emitting electrochemical cell using 4,7-Diphenyl-1,10-phenanthroline-bis(2-phenylpyridine)iridium(III) Hexafluorophosphate. J. Am. Chem. Soc..

[26] Bolink HJ, Coronado E, Costa RD, Lardiés N, Ortí E (2008). Near-quantitative internal quantum efficiency in a light-emitting electrochemical cell. Inorg. Chem..

[27] Costa, R. D. et al. Efficient deep-red light-emitting electrochemical cells based on a perylenediimide-iridium-complex dyad. *Chem. Commun*. 3886–3888 (2009). 10.1039/b905367k 19662241

[28] Wu H-B, Chen H-F, Liao C-T, Su H-C, Wong K-T (2012). Efficient and color-stable solid-state white light-emitting electrochemical cells employing red color conversion layers. Org. Electron..

[29] Zhang J (2013). Efficient light-emitting electrochemical cells (LECs) based on ionic iridium(III) complexes with 1,3,4-oxadiazole ligands. Adv. Funct. Mater..

[30] Reineke S (2009). White organic light-emitting diodes with fluorescent tube efficiency. Nature.

[31] Mesta M (2013). Molecular-scale simulation of electroluminescence in a multilayer white organic light-emitting diode. Nat. Mater..

[32] Su H-C, Wu C-C, Fang F-C, Wong K-T (2006). Efficient solid-state host-guest light-emitting electrochemical cells based on cationic transition metal complexes. Appl. Phys. Lett..

[33] Chen F-C, Yang Y, Pei Q (2002). Phosphorescent light-emitting electrochemical cell. Appl. Phys. Lett..

[34] Liao C-T, Chen H-F, Su H-C, Wong K-T (2012). Improving the balance of carrier mobilities of host-guest solid-state light-emitting electrochemical cells. Phys. Chem. Chem. Phys..

[35] Pertegas A (2014). Host-guest blue light-emitting electrochemical cells. J. Mater. Chem. C.

[36] Akatsuka T, Roldán-Carmona C, Ortí E, Bolink HJ (2014). Dynamically doped white light emitting tandem devices. Adv. Mater..

[37] Tang S, Buchholz HA, Edman L (2015). On the selection of a host compound for efficient host-guest light-emitting electrochemical cells. J. Mater. Chem. C.

[38] Tang S, Buchholz HA, Edman L (2015). White light from a light-emitting electrochemical cell: controlling the energy-transfer in a conjugatedpolymer/triplet-emitter blend. ACS Appl. Mater. Interfaces.

[39] Zeng Q, Li F, Guo T, Shan G, Su Z (2016). Large size color-tunable electroluminescence from cationic iridium complexes-based light-emitting electrochemical cells. Sci. Rep..

[40] Su H-C (2008). Solid-state white light-emitting electrochemical cells using iridium-based cationic transition metal complexes. J. Am. Chem. Soc..

[41] He L (2009). Toward highly efficient solid-state white light-emitting electrochemical cells: blue-green to red emitting cationic iridium complexes with imidazole-type ancillary ligands. Adv. Funct. Mater..

[42] He L (2010). Highly efficient blue-green and white light-emitting electrochemical cells based on a cationic iridium complex with a bulky side group. Chem. Mater..

[43] Su H-C, Chen H-F, Shen Y-C, Liao C-T, Wong K-T (2011). Highly efficient double-doped solid-state white light-emitting electrochemical cells. J. Mater. Chem..

[44] Su H-C (2012). Efficient solid-state white light-emitting electrochemical cells based on phosphorescent sensitization. J. Mater. Chem..

[45] Jhang Y-P (2013). Improving device efficiencies of solid-state white light-emitting electrochemical cells by adjusting the emissive-layer thickness. Org. Electron..

[46] Liao C-T, Chen H-F, Su H-C, Wong K-T (2011). Tailoring balance of carrier mobilities in solid-state light-emitting electrochemical cells by doping a carrier trapper to enhance device efficiencies. J. Mater. Chem..

[47] Tang S, Edman L (2010). Quest for an appropriate electrolyte for high-performance light-emitting electrochemical cells. J. Phys. Chem. Lett..

[48] Pina J, Seixas de Melo J, Burrows HD, Monkman AP, Navaratnam S (2004). On the triplet state of poly(N-vinylcarbazole). Chem. Phys. Lett..

[49] Fan C (2015). High-efficiency phosphorescent hybrid organic–inorganic light-emitting diodes using a solution-processed small-molecule emissive layer. ACS Appl. Mater. Interfaces.

[50] Takeshi Y, Yoshihisa Y, De-Chun Z, Tetsuo T (2002). Carrier mobilities in organic electron transport materials determined from space charge limited current. Jap. J. Appl. Phys..

[51] Wagberg T (2008). On the limited operational lifetime of light-emitting electrochemical cells. Adv. Mater..

[52] Shavaleev NM (2013). Pulsed-current vs. constant-voltage light-emitting electrochemical cells with trifluoromethyl-substituted cationic iridium(III) complexes. J. Mater. Chem. C.

[53] Tordera D (2013). Low current density driving leads to efficient, bright, and stable green electroluminescence. Adv. Energy Mater..

[54] Valeur, B. Molecular Fluorescence: Principles and Applications. Wiley-VCH Verlag GmbH (2001).

[55] van Reenen S (2010). A unifying model for the operation of light-emitting electrochemical cells. J. Am. Chem. Soc..

