# Author Correction: Design rules for light-emitting electrochemical cells delivering bright luminance at 27.5 percent external quantum efficiency

**DOI:** 10.1038/s41467-020-15417-3

**Published:** 2020-03-31

**Authors:** Shi Tang, Andreas Sandström, Petter Lundberg, Thomas Lanz, Christian Larsen, Stephan van Reenen, Martijn Kemerink, Ludvig Edman

**Affiliations:** 1 The Organic Photonics and Electronics Group, Department of Physics, Umeå University, Umeå, SE-901 87 Sweden; 2 LunaLEC AB, Linnaeus väg 24, Umeå, SE-901 87 Sweden; 3 Complex Materials and Devices, Department of Physics, Chemistry and Biology (IFM), Linköping University, Linköping, SE-58183 Sweden

**Keywords:** Electronic devices, Organic LEDs

Correction to: *Nature Communications* 10.1038/s41467-017-01339-0, published online 30 October 2017.

The original version of this Article contained two errors in Fig. 2h, in which the photoluminescence spectra curve of the “PVK:OXD-7” was plotted incorrectly and the figure legends were incorrectly given. The correct version is:

which replaces the previous incorrect version:

This has now been corrected in both the PDF and HTML versions of the Article.

