# Supplementary material for: Design rules for light-emitting electrochemical cells delivering bright luminance at 27.5 percent external quantum efficiency
Source: Nat Commun. 2017 Oct 30;8:1190. doi: 10.1038/s41467-017-01339-0 (PMC5662711; doi:10.1038/s41467-017-01339-0)
Supplement: Supplementary file 1 — Supplementary Information [file 41467_2017_1339_MOESM1_ESM.pdf]

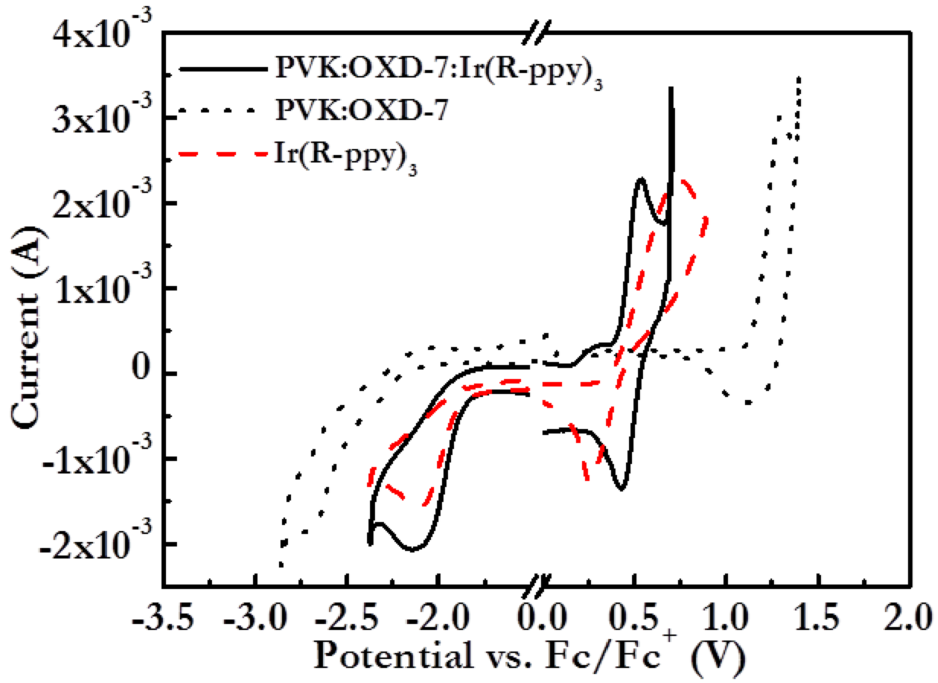

Supplementary Figure 1. | Cyclic voltammetry study of the host-guest compounds. CV traces of a PVK:OXD-7:Ir(R-ppy)$_3$ film (solid black line), a PVK:OXD-7 film (dotted black line), and a Ir(R-ppy)$_3$ film (dashed red line). The CV trace of the Ir(R-ppy)$_3$ thin film is similar to that of a PVK:OXD-7:Ir(R-ppy)$_3$ film, but distinctly different than the PVK:OXD-7 film void of the Ir(R-ppy)$_3$ compound. The conclusion is thus that Ir(R-ppy)$_3$ compound can be both p- and n-type doped in the PVK:OXD-7:Ir(R-ppy)$_3$ active-material film.

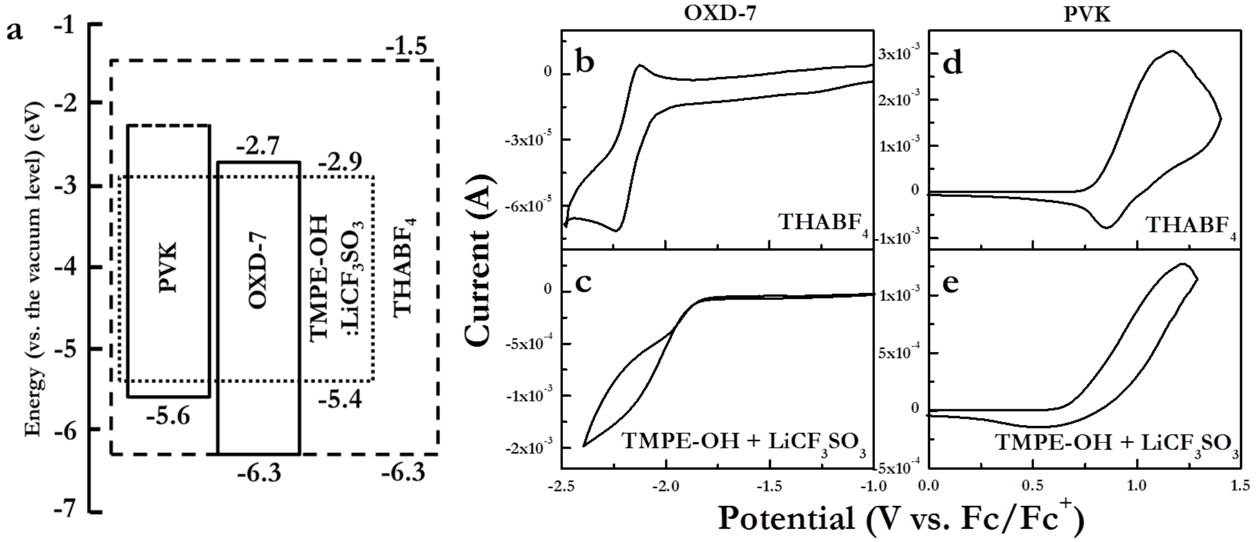

Supplementary Figure 2. | Electrochemical stability and doping capacity of the electrolytes. (a) The electron-energy levels of the host compounds and the two electrolytes. (b-c) CV traces in the cathodic regime for an OXD-7 film, using either (b) $THABF_4$ or (c) TMPE-OH:$LiCF_3SO_3$ as the electrolyte. (d-e) CV traces in the anodic regime for a PVK film, using either (d) $THABF_4$ or (e) TMPE-OH:$LiCF_3SO_3$ as the electrolyte. The electron-energy data show that the $THABF_4$ ionic liquid, but not the TMPE-OH:$LiCF_3SO_3$ electrolyte, displays a broad electrochemical stability window that encompasses the p- and n-type doping potentials of the PVK and OXD-7 host compounds. The CV data confirm this statement, since they show that is possible to electrochemically n-type dope OXD-7 and p-type dope PVK with $THABF_4$, but not with TMPE-OH:$LiCF_3SO_3$, as the supporting electrolyte.

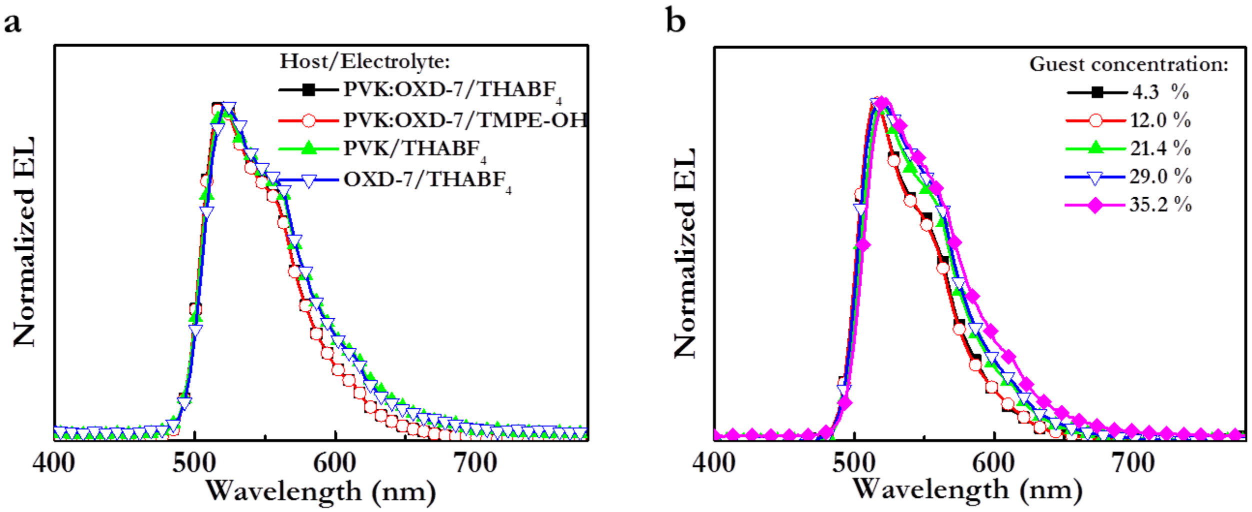

Supplementary Figure 3. | The EL spectra of the host-guest LEC devices. (a) The EL spectra of ITO/PEDOT:PSS/Host:Ir(R-ppy)$_3$:electrolyte/Al LECs, with the host and electrolyte selection identified in the inset. (b) The EL spectra of ITO/PEDOT:PSS/PVK:OXD-7:Ir(R-ppy)$_3$:THABF$_4$/Al LECs at different guest concentrations, as specified in the inset.

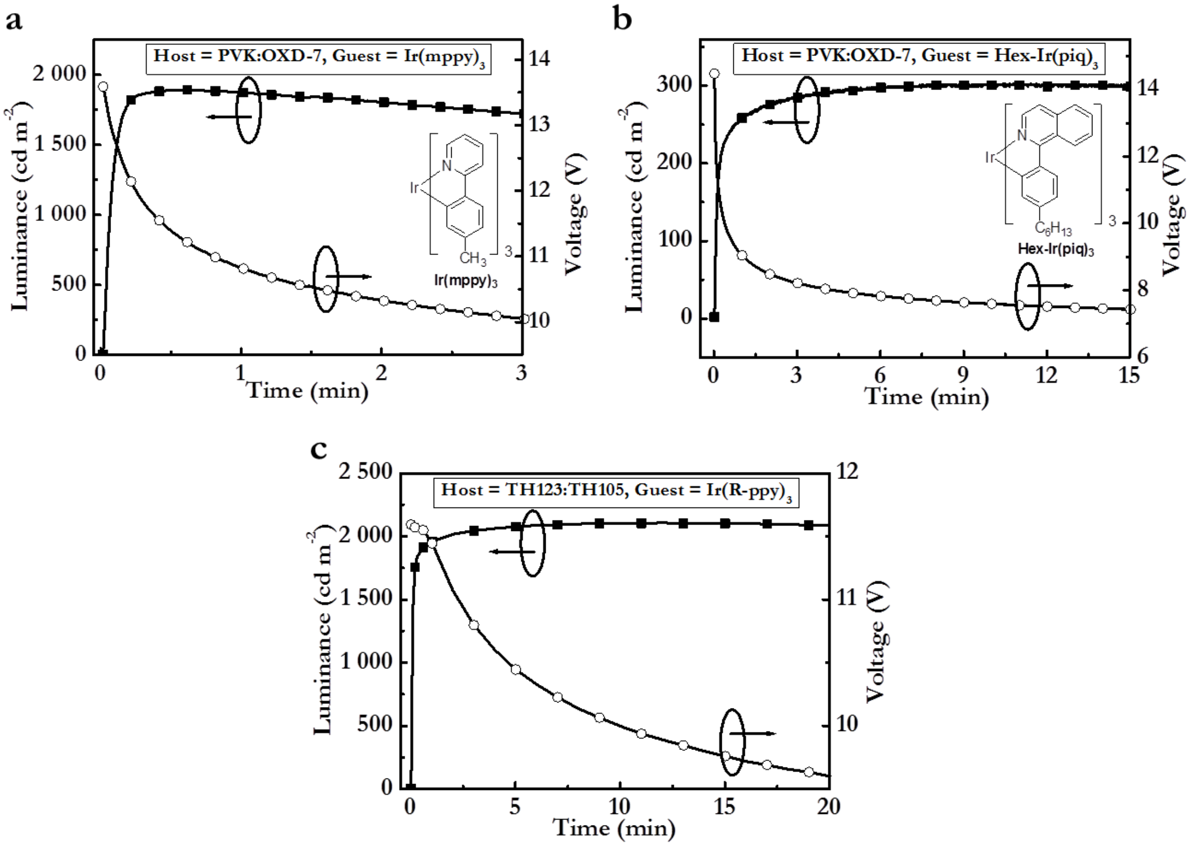

Supplementary Figure 4. **| Device performance of the three alternative high-performance host-guest LECs.** The temporal optoelectronic response of ITO/PEDOT:PSS/Host:guest:$THABF_4$/Al LECs with the host and guest selection defined in the inset. All devices featured optimized host:guest:electrolyte mass stoichiometry, identical active-material thickness (130 nm), and were drive by the same current density (j = 7.7 mA cm^{-2}). All host-guest LECs featured the same $THABF_4$ electrolyte concentration of 6.4 %, and the host-guest ratio had been systematically optimized for peak performance. Information on bipolar doping capacity, mobility, and trap depths are available in Supplementary Table 1.

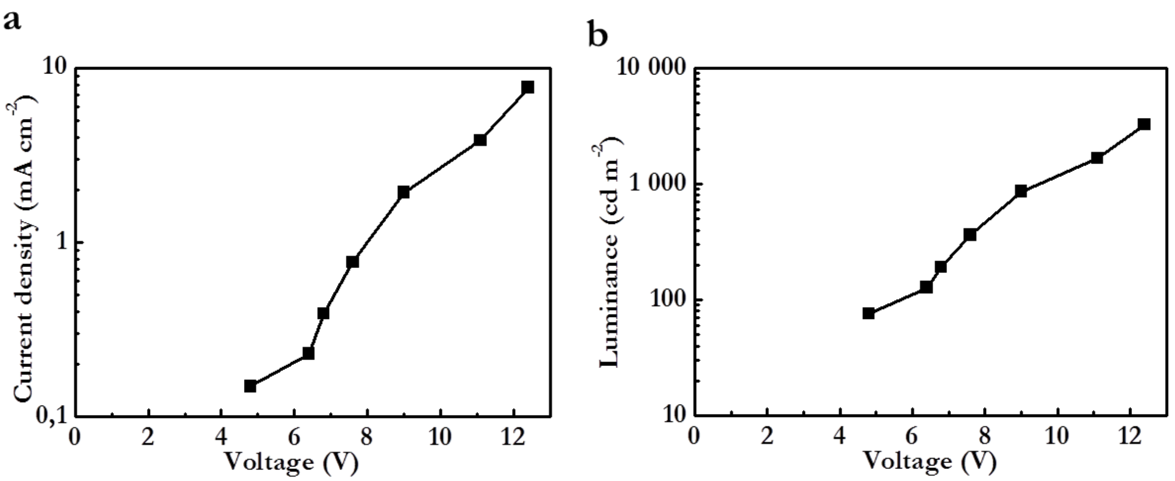

Supplementary Figure 5. │ The steady-state current and luminance as a function of voltage. The steady-state current density (a) and luminance (b) as a function of applied voltage for an ITO/PEDOT:PSS/PVK:OXD-7:Ir(R-ppy)$_3$:THABF$_4$/Al LEC with a guest concentration of 29 % and an electrolyte concentration of 6.4 %. The data were recorded at steady-state when the current and luminance had stabilized after the initial turn-on process. Both the current density and the luminance feature a close to logarithmic dependence on the applied voltage, and the onset potential for light emission is close to the effective energy-gap potential, as defined by the difference between the lowest LUMO and the highest HOMO of the PVK:OXD-7 host blend. The observation that the current density and luminance curves exhibit a similar gradient is in agreement with that the current efficacy is highly independent on the drive voltage.

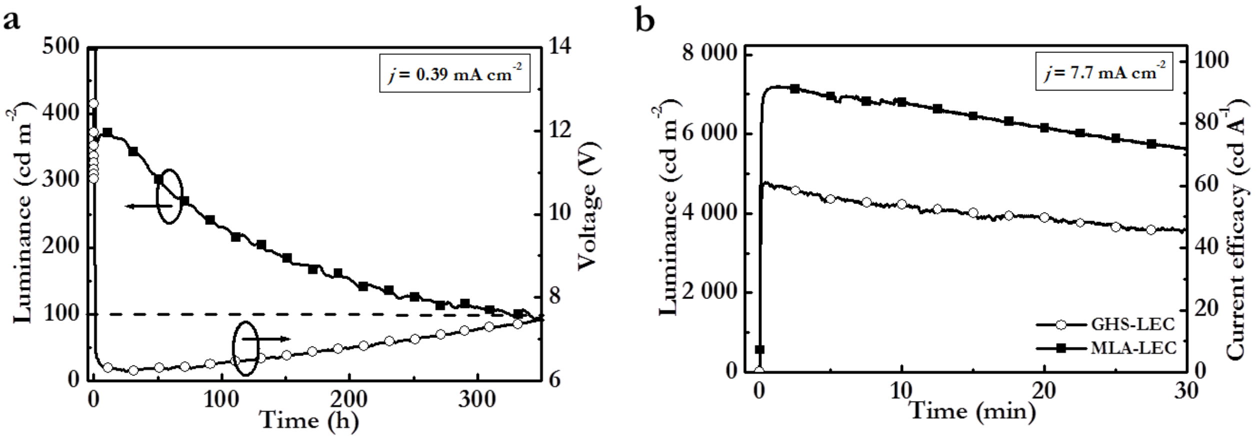

Supplementary Figure 6. | The stability of host-guest LECs. (a) The long-term operation of a GHS-LEC driven by j = 0.39 mA cm^{-2}. (b) The initial temporal evolution of the luminance and the current efficacy of the GHS-LEC (solid squares) and the MLA-LEC (open circles) driven by a current density of j = 7.7 mA cm^{-2}. More information on the performance metrics of these devices is tabulated in Supplementary Table 2.

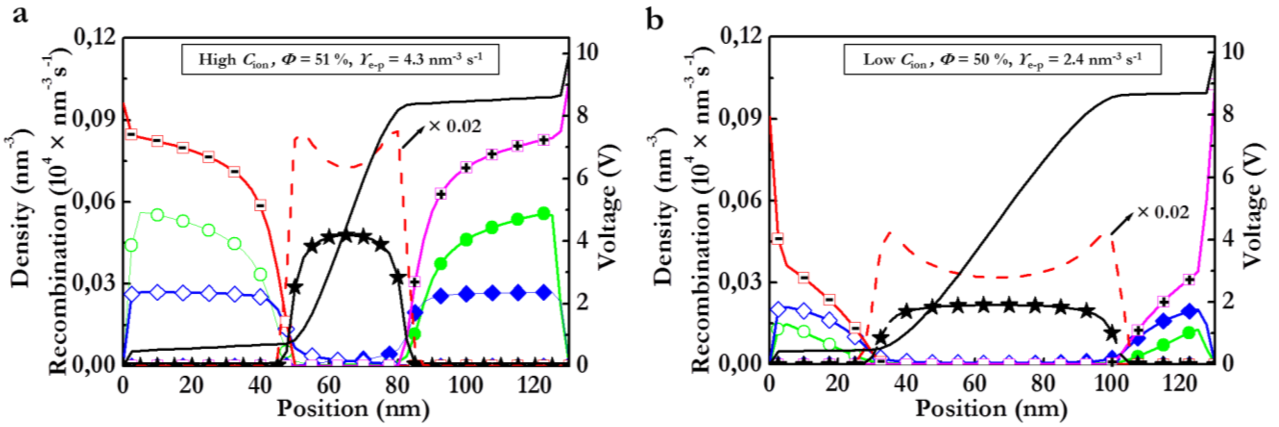

Supplementary Figure 7. │ Simulated steady-state profiles as a function of ion concentration. The simulated steady-state concentration and voltage profiles for a symmetric host-guest LEC at the original high ion concentration (a) and when the ion concentration has been lowered by a factor of three (b). The symbols are identified in Fig. 1c, with the exception of the exciton-polaron quenching rate which is indicated by the dashed red line. The data reveal that the steady-state spatial distribution of polarons (green and blue symbols) and the width of the light-emitting p-n junction (indicated by the black stars) can be controlled by the selected ion concentration. Specifically, with a lowering of the ion concentration, the p-n junction region width is increased when the distinct polaron distributions are gradually pushed towards the electrode interfaces, with an important consequence being that the peak exciton-polaron interaction rate ($\gamma_{e\text{-}p}$) drops significantly. With these data at hade, we could identify a "sweet-spot" ion concentration device (Supplementary Figure 7b), for which the electron-to-photon quantum efficiency Φ (see inset) and the voltage/current are essentially maintained while $\gamma_{e\text{-}p}$ is lowered by a factor of two (from 4.3 to 2.4 nm^{-3} s^{-1}) in comparison to the device presented in Supplementary Figures 7a and Figure 1d.

Supplementary Table 1. Performance summary of selected high-performance host-guest LECs

Host	Guest	Electrolyte	CIE (x, y)	Bipolar doping	Trap symmetry (E^{n}_{trap}/ E^{p}_{trap})	Mobility symmetry	Luminance (cd m^{-2})	Current efficacy at peak luminance (cd A^{-1})	EQE (%)
PVK:OXD-7	Ir(R-ppy)$_3$	THABF$_4$	0.30, 0.60	Yes	0.32/0.38	Yes	3,250	42.2	11.7
PVK	Ir(R-ppy)$_3$	THABF$_4$	0.30, 0.60	No	---/0.38	No	575	7.5	2.1
OXD-7	Ir(R-ppy)$_3$	THABF$_4$	0.30, 0.60	Yes	0.32/1.06	No	290	3.7	1.0
PVK:OXD-7	Ir(R-ppy)$_3$	TMPE-OH: LiCF$_3$SO$_3$	0.30, 0.60	No	0.32/0.38	Yes	315	4.0	1.1
PVK:OXD-7	Ir(mppy)$_3$	THABF$_4$	0.32, 0.63	Yes	0.06/0.23	Yes	1,890	24.6	6.9
PVK:OXD-7	Hex-Ir(piq)$_3$	THABF$_4$	0.68, 0.32	Yes	0.14/0.54	Yes	302	3.9	4.9
TH123:TH105	Ir(R-ppy)$_3$	THABF$_4$	0.30, 0.60	Yes	0.33/0.57	Yes	2,100	27.4	7.6

Supplementary Table 2. Performance of LEC devices equipped with external outcoupling structure.

Light outcoupling	Current efficacy (cd/A) (@luminance)	Power conversion efficacy (lm/W)	EQE (%)	Current efficacy (cd A^{-1}) (@luminance)	Power conversion efficacy (lm W^{-1})	EQE (%)
	j = 7.7 mA cm^{-2}			j = 1.9 mA cm^{-2}		
None	42.2 (3,250)	10.5	11.7	44.9 (864)	15.4	12.4
MLA	60.9 (4,700)	16.5	16.9	66.8 (1,285)	24.2	18.5
GHS	92.8 (7,200)	25.6	25.7	99.2 (1,910)	34.9	27.5

Supplementary Table 3. The performance of the symmetric host-guest LEC (guest concentration = 29 %) at different electrolyte concentration and drive current modes. The pulsed drive current mode featured a square-wave pulse with a frequency of 250 Hz and a duty cycle of 50 %.

Electrolyte concentration (%)	Drive current (mA cm^{-2})	Current efficacy (cd A^{-1}) (@ luminance)	Power conversion efficacy (lm W^{-1})	Steady-state voltage (V)	Lifetime (h) (@ luminance)
9.4	7.7 (constant)	36.8 (2870)	9.6	12.3	0.6 (>1000)
6.4	7.7 (constant)	42.2 (3250)	10.5	12.7	3.5 (>1000)
3.3	7.7 (constant)	40.0 (3077)	9.7	13.0	7.1 (>1000)
2.0	7.7 (constant)	34.1 (2621)	7.2	14.6	13 (>1000)
2.0	0.77 (constant)	40.1 (308)	11.7	10.7	300 (>100)
2.0	7.7 (pulsed)	30.8 (2390)	7.9	12.2	21 (>1000)

Supplementary Note 1

The parameters used in the simulations of the device in Figure 1d are given below. The corresponding experimental values are given in the second column when available. Typically somewhat lower concentrations for ions, traps *etc.* are used to reflect the fact that in the real device not all salt molecules will dissociate due to the ionic binding, nor that all guest molecules will cause a trap due to, *e.g.*, aggregation.

Simulation	*Experiment / comment*
$c_{ion} = 2 \times c_{salt} = 0.06$ (nm^{-3})	0.18 (nm^{-3})
$c_{trap} = 0.03$ (nm^{-3})	0.02→0.18 (nm^{-3}); 0.15 (nm^{-3}) (best experiment)
Ion/trap ratio = 2	1.2
$c_{host} = 0.3$ (nm^{-3})	≈1.5 (nm^{-3}) (best experiment)
Trap/DOS ratio = 10 %	≈10 % (best experiment)
$E_{trap}^{n} = E_{trap}^{p} = 0.10$ eV	0.3-0.4 eV (from CV). Simulation value set lower to reflect disorder-assisted escape of the electronic charge from its counter ion.
Active-material thickness = 130 nm	130 nm
LUMO/HOMO = 2.7/5.6 eV	LUMO (OXD-7): 2.7 eV, HOMO (PVK): 5.6 eV
$E_{F,contact}$ (LEC) = 4.2/5.0 eV	Al cathode: 4.1-4.2 eV, PEDOT:PSS anode: ≈5.0 eV
$E_{F,contact}$ (OLED) = 2.9/5.0 eV	Ca cathode: 2.9 eV, PEDOT:PSS anode: ≈5.0 eV
$\varepsilon_r = 3.6$	
Free n/p mobility (host, LEC) = 1×10^{-12} m^2 (Vs)$^{-1}$	

Free n/p mobility (host, OLED) = 1×10^{-10} m^2 $(Vs)^{-1}$

Ion mobility = 1×10^{-13} m^2 $(Vs)^{-1}$

Irrelevant for steady-state solution; one order slower than electronic mobility selected to be able to observe different time scales in transients

Exciton radiative decay rate: 1.0 μs^{-1} (Ref. [1])

$D_{ex} = 4.6 \times 10^{-11}$ m^2 s^{-1} (trap-free) Corresponds to a diffusion length of 6.8 nm (Ref. [2])

Förster critical radius R_0 = 1.5 nm (Ref. [1])

Supplementary References

1. Mesta M, *et al*. Molecular-scale simulation of electroluminescence in a multilayer white organic light-emitting diode. *Nat. Mater*. **12**, 652-658 (2013).

2. van Reenen S, Janssen RAJ, Kemerink M. Fundamental Tradeoff between Emission Intensity and Efficiency in Light-Emitting Electrochemical Cells. *Adv. Funct. Mater*. **25**, 3066-3073 (2015).
